# spillR: spillover compensation in mass cytometry data

**DOI:** 10.1093/bioinformatics/btae337

**Published:** 2024-06-06

**Authors:** Marco Guazzini, Alexander G Reisach, Sebastian Weichwald, Christof Seiler

**Affiliations:** Department of Advanced Computing Sciences, Maastricht University, Maastricht, The Netherlands; MAP5, Université Paris Cité, CNRS, Paris, France; Department of Mathematical Sciences, University of Copenhagen, Copenhagen, Denmark; Department of Advanced Computing Sciences, Maastricht University, Maastricht, The Netherlands; Mathematics Centre Maastricht, Maastricht University, Maastricht, The Netherlands; Center of Experimental Rheumatology, Department of Rheumatology, University Hospital Zurich, University of Zurich, Schlieren, Switzerland

## Abstract

**Motivation:**

Channel interference in mass cytometry can cause spillover and may result in miscounting of protein markers. Chevrier *et al.* introduce an experimental and computational procedure to estimate and compensate for spillover implemented in their R package CATALYST. They assume spillover can be described by a spillover matrix that encodes the ratio between the signal in the unstained spillover receiving and stained spillover emitting channel. They estimate the spillover matrix from experiments with beads. We propose to skip the matrix estimation step and work directly with the full bead distributions. We develop a nonparametric finite mixture model and use the mixture components to estimate the probability of spillover. Spillover correction is often a pre-processing step followed by downstream analyses, and choosing a flexible model reduces the chance of introducing biases that can propagate downstream.

**Results:**

We implement our method in an R package spillR using expectation-maximization to fit the mixture model. We test our method on simulated, semi-simulated, and real data from CATALYST. We find that our method compensates low counts accurately, does not introduce negative counts, avoids overcompensating high counts, and preserves correlations between markers that may be biologically meaningful.

**Availability and implementation:**

Our new R package spillR is on bioconductor at bioconductor.org/packages/spillR. All experiments and plots can be reproduced by compiling the R markdown file spillR_paper.Rmd at github.com/ChristofSeiler/spillR_paper.

## 1 Introduction

Mass cytometry makes it possible to count a large number of proteins simultaneously on individual cells (Bandura *et al.* 2009, Bendall *et al.* 2011). Although mass cytometry has less spillover—measurements from one channel overlap less with those of another—than flow cytometry (Bagwell and Adams 1993, Novo *et al.* 2013), spillover is still a problem and affects downstream analyses such as differential testing (Weber *et al.* 2019, Seiler *et al.* 2021) or dimensionality reduction (McCarthy *et al.* 2017). Reducing spillover by careful design of experiment is possible (Takahashi *et al.* 2017), but a purely experimental approach may be neither efficient nor sufficient (Lun *et al.* 2017).

Chevrier *et al.* (2018) propose a method for addressing spillover by conducting an experiment on beads. This experiment measures spillover by staining each bead with a single type of antibody. The slope of the regression line between target antibodies and non-target antibodies represents the spillover proportion between channels. Miao *et al.* (2021) attempt to solve spillover by fitting a mixture model. Our contribution combines the solutions of Chevrier *et al.* (2018) and Miao *et al.* (2021). We still require a bead experiment, as in Chevrier *et al.* (2018), but estimate spillover leveraging a statistical model, as in Miao *et al.* (2021). Both previous works rely on an estimate for the spillover matrix, which encodes the pairwise spillover proportion between channels. We avoid estimating a spillover matrix and instead model spillover by fitting a mixture model to the counts observed in the bead experiment. Our main new assumption is that the spillover distribution—not just the spillover proportion—from the bead experiment carries over to the biological experiment. In other words, we transfer the spillover distribution to the real experiment instead of just the spillover proportion encoded in the spillover matrix.

In Section 2, we present our mixture model and link it to calculating spillover probabilities for specific count values. Our estimation procedure is based on an EM algorithm, and implemented in our new R package spillR. In Section 3, we conduct experiments on simulated, semi-simulated, and real data obtained from the CATALYST R package (Chevrier *et al.* 2018). Section 4 discusses our experiments and relates our findings to CATALYST.

## 2 Materials and methods

In this section, we first illustrate our method spillR (as well as a simple baseline spillR-naive) by an example and then describe the algorithm and its underlying assumptions. Regarding terminology, mass cytometry counts are often referred to as dual counts or signal intensity; we refer to them as counts to emphasize their nature as non-negative integers, as opposed to possibly real-valued intensities.

### 2.1 Example

**Figure 1.**
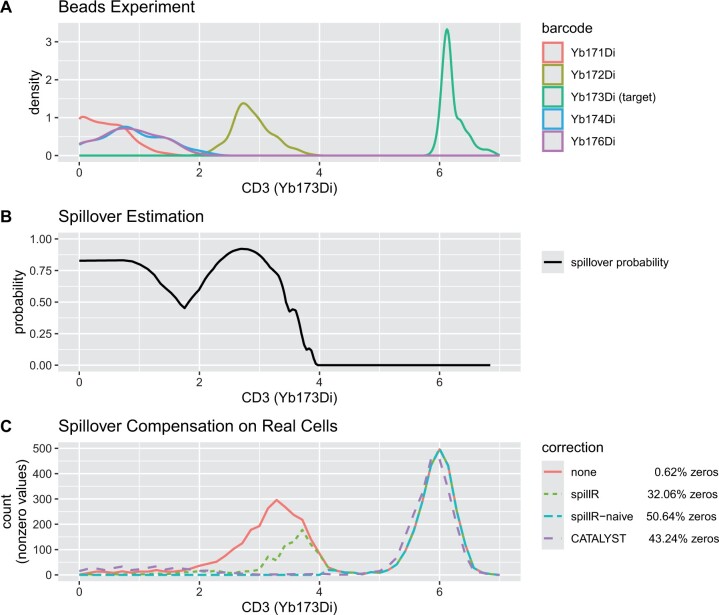
Panel (A) shows a density plot of target and spillover markers based on the beads experiment, Panel (B) shows spillover probability for Yb173Di estimated by spillR, and Panel (C) compares spillover compensation on real cells by our methods and CATALYST. Counts are arcsinh transformed with cofactor of five (Bendall *et al.* 2011), zero counts are not shown. As seen in Panel (C), our baseline method spillR-naive performs similarly to CATALYST and compensates the first peak of the uncorrected data (red) between about 2 and 4 as spillover. By contrast, spillR is sensitive to the difference in shape between the peaks in the bead data (A) and the first peak in the real data (C, red) and only compensates the part of the red curve as spillover that matches the bead experiment. This figure is an example of the diagnostic plot obtained when using the function plotDiagnostics in spillR

Figure 1 illustrates our procedure using a dataset from the CATALYST package as an example. There are four markers, HLA-DR (Yb171Di), HLA-ABC (Yb172Di), CD8 (Yb174Di), and CD45 (Yb176Di), that spill over into the target marker, CD3 (Yb173Di). The markers have two names: the first name is the protein name and the second name in brackets is the conjugated metal. There are bead experiments for each of the spillover markers.

Panel A depicts the marker distributions from the beads experiment. We see that for this marker the bead experiments are high-quality as the target marker Yb173Di is concentrated around six, similarly to the experiment with real cells. This suggests that the spillover marker values can be transferred to the real experiments. Marker Yb172Di shows large spillover into Yb173Di and suggests that the left tail of the first mode of the distribution observed on real cells may be attributed to that marker. The other spillover markers have low counts in the bead experiment, making it justifiable to set some or all of the low counts on real cells to zero.

Panel B shows a curve representing our spillover probability estimates. We can see that the probability of spillover is high for counts with a high density of spillover markers (panel A) and a low density of the target marker (panel C). If the spillover probability is close to one, our correction step assigns most cells to spillover. Counts above 4 stem from spillover with probability zero (and from the actual target with probability one), which means that our procedure keeps them at their raw uncorrected value.

Panel C displays the distribution of our target marker, CD3 (Yb173Di), before and after spillover correction. We observe few real counts (red) below 2, so although all methods perform strong compensation in this range, there is little visible difference between uncompensated and compensated counts. Above 2 there is a clear distinction between the compensation methods. CATALYST, like our baseline spillR-naive, compensates nearly all counts forming the first peak of the raw counts (red, between 2 and 4) as spillover. By contrast, spillR compensates only where the density of spillover markers in the bead experiment shown in Panel A is high (e.g. Yb172Di spillover peaks at around 2.7). As a result, it does not compensate for all of the counts forming the first peak of the red curve and compensates more counts between 2 and 3 than between 3 and 4. While CATALYST shifts large counts (around 6) slightly to the left, our methods leave them unchanged as the bead experiment shows no spillover in this range. Our baseline method spillR-naive is similar to CATALYST in the low and medium ranges but keeps higher counts unchanged.

### 2.2 Definition of spillover probability and assumptions

We observe a count *Y_i_* of a target marker in cell *i*. We model the observed *Y_i_* as a finite mixture (McLachlan *et al.* 2019) of unobserved true marker counts $Y_{i}\;|\;Z_{i}=1$ and spillover marker counts $Y_{i}\;|\;Z_{i}=2,\ldots ,Y_{i}|Z_{i}=K$ with mixing probabilities $\pi_{k}=P(Z_{i}=k)$ for k=1,…,K,

$$P(Y_{i}=y)=\sum_{k=1}^{K}\pi_{k}P(Y_{i}=y|Z_{i}=k).$$

The mixing probability *π*_1_ is the proportion of true signal in the observed counts. The other *K −* 1 mixing probabilities are the proportions of spillover. The total sum of mixing probabilities equals one, $\sum_{k}\pi_{k}=1$. The total number of markers in mass cytometry panels is between 30 and 40 (Bendall *et al.* 2011), but only a small subset of three to four markers spill over into the target marker (Chevrier *et al.* 2018). So, typically *K *=* *1 + 3 or *K *=* *1 + 4.

Experimentally, we only measure samples from the distribution of *Y_i_*. The probabilities *π_k_* and true distributions $P(Y_{i}=y\;|\;Z_{i}=k)$ are unobserved, and we need to estimate them from data. In many applications, the mixture components are modeled to be in a parametric family, e.g. the negative binomial distribution. As spillover correction is a pre-processing step followed by downstream analyses, choosing the wrong model can introduce biases in the next analysis step. To mitigate such biases, we propose to fit nonparametric mixture components. We make two assumptions that render the components and mixture probabilities identifiable:

- (A1) Spillover distributions are the same in bead and real experiments.
  The distribution of $Y_{i}\;|\;Z_{i}=k$ for all *k *>* *1 is the same in beads and real cells. This assumption allows us to learn the spillover distributions of $Y_{i}\;|\;Z_{i}=k$ for all *k *>* *1 from experiments with beads, and transfer them to the experiment with real cells. This assumption relies on high-quality single-stained bead experiments that measure spillover in the same range as the target biological experiment. In other words, a bead experiment for our method works best if the distribution of bead cells is similar to the distribution of real cells.
- (A2) For each cell *i*, the observed count *Y_i_* can only be due to one marker.
  This assumption is already implied by the statement of the mixture model. It allows us to calculate the spillover probability for a given count *Y_i_* = *y* from the posterior probability that it arises through spillover from markers *k *>* *1,
  $$\begin{array}{cc} P(\text{spillover}|Y_{i}=y) & =P(Z_{i}>1|Y_{i}=y)\\ & =1-P(Z_{i}=1|Y_{i}=y)\\ & =1-\frac{\pi_{1}P(Y_{i}=y|Z_{i}=1)}{P(Y_{i}=y)}. \end{array}$$
  To parse this calculation, recall that in mixture models the *π*_1_ is the prior probability, $P(Y_{i}=y|Z_{i}=1)$ is the conditional probability given the mixture component, and the denominator $P(Y_{i}=y)$ is the marginal distribution. Applying Bayes rule leads to the posterior probability.

### 2.3 Estimation of spillover probability

We propose a two-step procedure for estimating the spillover probability. In step 1, we estimate mixture components and mixture probabilities. We refine these estimates using the EM algorithm (Dempster *et al.* 1977). In step 2, we use these probability estimates to assign counts to spillover or target marker signal.

We denote the *n *×* K* count matrix as $Y=(y_{ik})$ with real cells in the first column and beads in columns two and higher. To simplify mathematical notation but without loss of generality, we assume that the number of events from real and bead experiments have the same *n*. In practice, the number of events from bead experiments is much smaller than from real experiments. For *k *>* *1, the *k*th column of **Y** contains marker counts for a given spillover marker, which represents the empirical spillover distribution of marker *k* into the target marker represented by the first column of **Y**.

#### 2.3.1 EM algorithm

- Initialization: For the mixture probability vector, we assign probability 0.9 to the target marker and divide the probability 0.1 among the spillover markers,
  $${\hat{\pi }}_{1}=0.9\;\text{and}\;{\hat{\pi }}_{i}=0.1/(K-1)\;\text{for all}\;i>1.$$
  The procedure is not sensitive to the choice of the initial mixture probability vector and other initializations are possible but may be slower to converge. Then, we initialize the *k*th mixture component using its probability mass function (PMF) after smoothing and normalizing, $\hat{P}(Y_{i}=y\;|\;Z_{i}=k)$. We smooth the PMF using kernel density estimation implemented in the R function density with the default option for selecting the bandwith of a Gaussian kernel.
- E-step: We evaluate the posterior probability of a count *y* belonging to component *k* (i.e. originating from marker *k*),
  $$\hat{P}\left(Z_{i}=k\;|\;Y_{i}=y\right)=\frac{{\hat{\pi }}_{k}\hat{P}(Y_{i}=y\;|\;Z_{i}=k)}{\sum_{k\prime =1}^{K}{\hat{\pi }}_{k\prime }\hat{P}(Y_{i}=y\;|\;Z_{i}=k\prime )}.$$
- M-step: We estimate the new mixture probability vector from posterior probabilities,
  $${\hat{\pi }}_{k}=\frac{1}{n}\sum_{i=1}^{n}\hat{P}\left(Z_{i}=k|Y_{i}=y\right),$$
  and estimate the new target marker distribution by smoothing and normalizing. Here, we use the R function density again, weighing each observation according to its posterior probability $\hat{P}\left(Z_{i}=1|Y_{i}=y\right)$. We only update the target marker distribution, $\hat{P}(Y_{i}=y|Z_{i}=1)$, and keep the other bead distributions, $\hat{P}(Y_{i}=y|Z_{i}=k)$ for all *k *>* *1, fixed at their initial value.

To refine our estimates, we iterate over the E and M-steps until estimates stabilize. We stop iterating when ${\hat{\pi }}_{1}$ changes less than $10^{-5}$ from the previous iteration. The final output is the spillover probability curve with estimates at discrete points in the support of *Y_i_*,

$$\hat{P}(\text{spillover}\;|\;Y_{i}=y)=1-\hat{P}(Z_{i}=1\;|\;Y_{i}=y).$$

We rely on assumption (A1) to justify updating only the distribution of the target marker. We rely on assumption (A2) to justify calculating the spillover probability from the mixture model. We refer to the Supplementary material for a step-by-step example of our EM algorithm.

#### 2.3.2 Spillover decision

To perform spillover compensation, we draw from a Bernoulli distribution with the spillover probability as parameter to decide whether or not to assign a given count to spillover. We mark counts attributed to spillover by setting them to a user-specified value. We recommend a value of zero to maintain the overall cellular composition of the sample, or a value such as NA or –1 to mark spillover counts for separate treatment in downstream analyses (e.g. calculating means only over non-spillover counts).

### 2.4 Baseline method

We compare our mixture method to a naive baseline method spillR-naive that considers only the bead distributions. We replace the real cells in the first component *k *=* *1 with their bead distribution. Similarly to our standard spillR method, we estimate the bead PMF of each bead *k* with the kernel density estimator density, $\hat{P}(Y_{i}=y|Z_{i}=k)$. Then, for all count values *y* in the range of the bead counts, we separately normalize the PMF at each value *Y _i_* = *y* and calculate the spillover probability as

$$\hat{P}(\text{spillover}\;|\;Y_{i}=y)=1-\frac{\hat{P}(Y_{i}=y\;|\;Z_{i}=1)}{\sum_{k\prime =1}^{K}\hat{P}(Y_{i}=y\;|\;Z_{i}=k\prime )}.$$

We proceed as in our standard spillR method to decide whether or not to assign a given count to spillover. This is a computationally efficient and simple baseline that assigns counts to markers in proportion to their density at that count value in the corresponding bead experiment.

## 3 Results

We first evaluate our new method spillR on simulated datasets. We probe our method to experimentally find its shortcomings. Then, we compare spillR to the non-negative least squares method implemented in the R package CATALYST on real and semi-simulated data from the same package.

### 3.1 Simulated data

**Figure 2.**
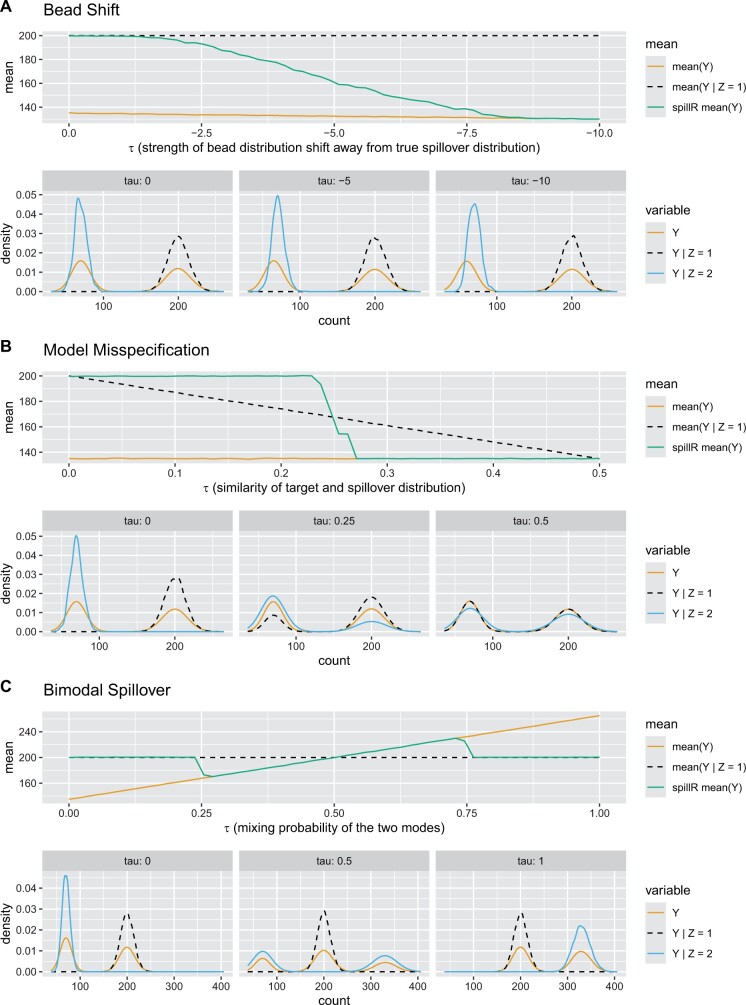
Three experiments testing our assumptions and sensitivity to bimodal bead distributions. For each experiment, the top row are mean values over the entire range of the experimental setups. The mean values for spillR are computed on values not marked as NA, so the mean ignores the counts attributed to spillover. The bottom row are density plots for three parameter settings to illustrate the generated distributions. *Y* is the distribution with spillover. Y|Z=1 is the distribution without spillover. Y|Z=2 is the spillover. mean(*Y*) is the average of the distribution with spillover. mean(Y|Z=1) is the average count without spillover. spillR mean(*Y*) is the average count after correcting *Y*

We choose three different experiments to test spillR under different bead and real cell distributions. We explore a wide range of possible parameter settings. Figure 2 has three panels, each representing one experimental setup. The first two panels test our assumptions (A1) and (A2). The third panel tests the sensitivity of spillR to bimodal bead distributions. For all three experiments, we model counts using a Poisson distribution with parameter *λ*. We simulate 10 000 real cells with *λ* = 200 and 1000 beads with *λ* = 70, and a spillover probability of 0.5. Unless otherwise specified, the bead data are drawn from the true spillover distribution. The other parameters and statistical dependencies are specific to each experiment. The details of the generative models are given in the Supplementary material. We repeat each simulation 20 times and report averages over the 20 replications.

Each panel of Fig. 2 has two rows of plots. The plot in the first row represents the summary of the means for each experimental setup as a function of their respective parameter *τ*. This parameter has a different meaning in each setup. To visualize the different experiments, we summarize the full distributions with the true simulated signal mean (black), the uncorrected mean (orange), and the spillR corrected mean (green). Plots on the second row illustrate the simulated data distributions for three selected parameters *τ* picked from the experimental setup. The yellow density curve shows the observed counts *Y*. The black density curve shows the distribution of target cell counts. The blue density curve shows the distribution of spillover counts. The yellow density curve represents the data *Y* we would observe in practice. We simulate this data using the models in the Supplementary material. The goal of the experiment is to estimate the mean of the true counts (black density curve) as accurately as possible from the observed counts (yellow density curve). Using NA imputation for spillover counts, the average of the compensated observed counts when ignoring NA values is equal to the mean of the true counts if all spillover counts are correctly identified as such.

In the first experiment (panel A), we shift the spillover in the beads experiment away from the true spillover to probe (A1). We test a range of bead shifts from no shift at *τ *= 0 to τ=−10. At τ=−10, the measured spillover (the first mode of the yellow density) is shifted away from the actual spillover (the blue density), causing both the observed and compensated mean to be lower than the true mean. This may be the case in a low-quality bead experiment. As *τ* gets closer to zero, the first mode of the yellow density moves toward the blue density (as may be the case in a higher quality bead experiment), and the compensated signal moves closer to the true mean.

In the second experiment (panel B), we mix target and spillover to explore the robustness of our method with respect to our second assumption (A2). One way to think about this is that the mixture is a form of model misspecification. Our mixture model is undercomplete, which means that there are more true mixture components than we observe in the beads experiment. If *τ *= 0, then assumption (A2) is correct, but for τ=0.5, the assumption (A2) is maximally violated. The true mean decreases with increasing *τ*. spillR compensates well as long as *τ* is close to zero but does not adapt immediately as the spillover distribution gets closer to the target distribution for increasing *τ*. When the mean of the spillover distribution crosses the mid-way point to that of the target marker distribution at τ≈0.25, the mean of counts compensated by spillR flips to the mean of the observed data, until at τ=0.5 all three distributions and their means are the same.

In the third experiment (panel C), we model spillover with a bimodal distribution. Here, *τ* is the mixing probability of the two modes. The locations of the two spillover modes are fixed. If *τ*  =  0 or *τ* = 1, then spillover is unimodal. If τ=0.5, the first mode of the bimodal bead distribution is left to the signal mode, and the second mode is to the right. The corrected mean is closer to the true mean than the uncorrected mean across the test range.

### 3.2 Real data

We compare our methods to CATALYST on one of the example datasets in the CATALYST package. The dataset consists of an experiment with real cells and corresponding single-stained bead experiments. The experiment on real cells has 5000 peripheral blood mononuclear cells from healthy donors measured on 39 channels. The experiment on beads has 10 000 cells measured on 36 channels with the number of beads per metal label ranging from 112 to 241.

**Figure 3.**
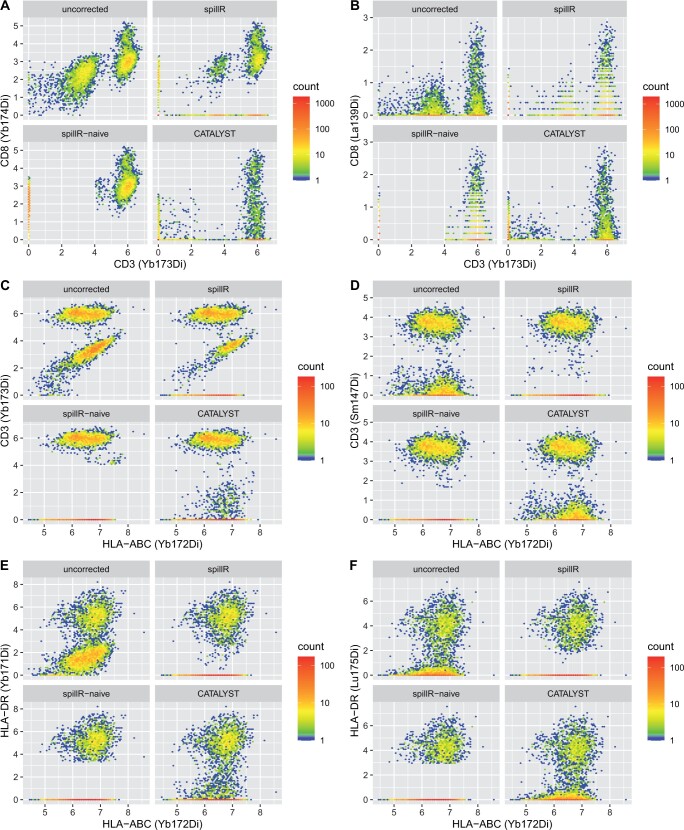
Comparison of compensation methods and uncorrected counts on real data. Counts are arcsinh transformed with cofactor of five (Bendall *et al.* 2011)

In Fig. 3, we show the comparison of our methods to CATALYST on the same markers as their original paper (Chevrier *et al.* 2018) in their Fig. 3B. In the original experiment, they conjugate the three proteins CD3, CD8, and HLA-DR with two different metal labels. For example, they conjugate CD8 with Yb174Di (Yb is the metal and the number indicates the number of nucleons of the isotope) and La139Di. As in their plot, our columns correspond to the different metal labels. In the first column are isotopes that are close to one another, which can cause spillover. In the second column are isotopes that are far from one another, which should not cause spillover. Each row represents a comparison for a target channel (on the horizontal axis) and the potential spillover channel (on the vertical axis). We visualize the joint distributions using two-dimensional histograms.

In all six panels (A–F), we observe that spillR compensates most strongly in the low counts, whereas CATALYST compensates strongly in the middle range. From the experimental setup, we expect strong spillover in the first column, and little spillover in the second column. In the first column, spillR seems to undercompensate in panels A and C, but compensates strongly in panel E, whereas we observe the opposite trend for CATALYST. In the second column, we find that CATALYST compensates substantially in panel B, and spillR compensates substantially in panels D and F, even though we expect little spillover. The baseline method spillR-naive can be seen to compensate strongly in all cases.

A characteristic pattern can be seen in panel C, CD3 (Yb173Di) against HLA-ABC (Yb172Di). CATALYST compensates strongly in the middle range and removes the spherical pattern that shows correlation between the two markers. spillR preserves this correlation structure and only compensates the lower counts of CD3 (Yb173Di). This highlights a key difference between spillR and CATALYST: spillR identifies counts that may arise from spillover and replaces them with a user-specified value (e.g. 0, NA, or −1), whereas CATALYST shrinks counts across the entire range to compensate for spillover.

The color code of the two-dimensional histograms indicates the absolute number of cells that fall into one hexagon bin. The uncorrected and spillR corrected histograms can contain different absolute numbers of cells, even for identical distributions. This is due to a rounding step in spillR that converts raw counts to integers. Raw mass cytometry data may not be true count data because the proprietary post-processing of the manufacturer often performs a randomization step when exporting the data. The uncorrected counts do not undergo this pre-processing step, and CATALYST does not perform this pre-processing step either. This also explains the different patterns in panel B. spillR has horizontal stripes that correspond to non-integer values not in the support of the distribution for spillR. We leave the decision to apply re-randomization of the count data for downstream analysis up to the user. Our rationale is that the user should see the differences in this pre-processing step and how it propagates to the results.

The average computation time for the experiment shown in Fig. 3 with 100 replications on an Apple M1 with 8 cores and 16 GB of RAM is 10.6 s for spillR, 0.43 s for CATALYST, and 0.45 s for spillR-naive. The computational costs scale linearly in the number of cells and the number of spillover markers. This allows for processing large-scale datasets.

### 3.3 Semi-simulated data

**Figure 4.**
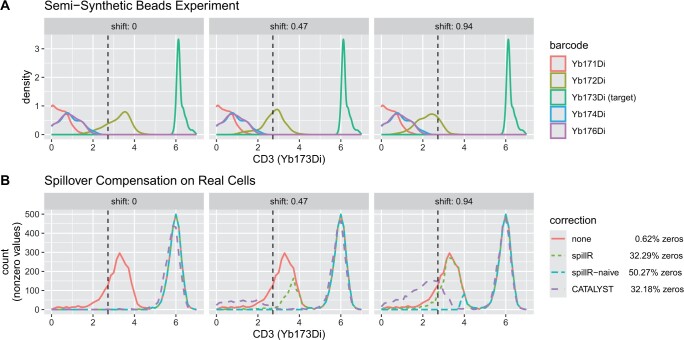
Comparison of compensation methods and uncorrected counts on semi-synthetic data (spillR and spillR-naive are set to impute spillover values with 0). The vertical dashed line helps to interpret the spillover correction. It indicates the original mode of the bead distribution of Yb172Di at 2.7, before overwriting it with the first peak of the real observations of Yb173Di. Counts are arcsinh transformed with cofactor of five (Bendall *et al.* 2011). The zero percentages are averages over all three experiments

We compare spillR and CATALYST on semi-simulated data in order to elucidate differences between spillR and CATALYST, and to evaluate the performance of spillR when more than one marker spills into the target marker. We create semi-simulated datasets by overwriting the bead distribution for the target marker CD3 (Yb173Di). We take the first mode of the count distribution of CD3 (Yb173Di) observed in real cells (the counts from 1.44 to 4.79 on the transformed scale) as a reference range. We overwrite the bead distribution of Yb172Di, which dominates this range, by the observed cell distribution in the same range with three different shift values: no shift is 0, subtracting 0.47 on the transformed scale, and subtracting 0.94 on the transformed scale. We further subsample without replacement from this new bead distribution to keep the same number of beads as in the original dataset. Figure 4 shows the three different beads experiment datasets in row A and the resulting compensations in row B.

In the first column of Fig. 4, the bead distributions are equal to the original dataset from Fig. 1 except Yb172Di is now perfectly aligned with the first mode of the distribution of real cells (red curve in row B). In the second and third column, we shift the bead distribution of Yb172Di by 0.47 and 0.94. All three methods correctly compensate the spillover mode when no shift is present (first column). CATALYST and spillR-naive compensate more aggressively in the medium shift cases (second column), while spillR is more moderate and compensates only the left-hand tail of the spillover mode. For a shift of 0.94 (third column), the three methods differ: CATALYST shrinks counts toward zero, shifting the entire spillover toward zero (resulting in many counts between about 1 and 3), spillR compensates lightly on the left-hand tail, and spillR-naive compensates aggressively leaving only a small right-hand tail. This experiment illustrates how spillR compensates most strongly for counts that can be attributed to spillover following the distribution observed in the beads experiment.

## 4 Discussion

The sensitivity analysis in Section 3.1 illustrates the performance of spillR under different conditions. The experiment for (A1) shows that the mean count after spillR correction is closer to the true mean over a wide range of bead shifts. This indicates that our method can perform well even if the bead experiments are imperfect. If the difference between distributions of beads and real cells is large, then one option is to rerun the bead experiments to reduce this gap. The experiment for (A2) shows that our method is robust to model misspecification. Additionally, misspecification can be addressed by adding all channels if necessary. The increase in computational cost when adding channels is relatively minor as our method scales linearly in the number of spillover markers. The experiment on bimodal bead distributions shows that the mean count after correction is still closer to the true mean even with bimodal bead distributions and even if the spillover is larger than the true signal.

In our comparison with CATALYST on real data (Section 3.2) and semi-simulated data (Section 3.3), we observe the effect of the two different correction strategies. CATALYST shrinks all counts toward zero by minimizing a non-negative least squares objective. It assumes that spillover is linear up to counts of 5000. The applied shrinkage is the same for low counts (e.g. below 10) and high counts (e.g. more than 100). By contrast, spillR does not require linearity of the spillover, but assumes that the distribution on the beads experiment carries over to the real cells experiment. If counts are in the spillover range (which mostly applies to low counts), they are corrected strongly and set to a user-specified imputation value. If counts are not in the spillover range, they are left unchanged. Among the unchanged counts, correlations between markers are preserved. The marker correlation between HLA-ABC (Yb172Di) and CD3 (Yb173Di) shown in the first column of Fig. 3 illustrates this point. CATALYST removes the positively correlated count concentration, whereas spillR keeps it. Compensation methods have to balance between compensating for spillover while keeping potentially biologically meaningful signals for unbiased downstream analyses. In this example, further experiments on the correlation structure between these markers would be necessary to resolve the discrepancy between the two methods. This is an important point as discovering correlations between markers can lead to the discovery of new clusters or signaling networks.

Our baseline method, spillR-naive, illustrates the behavior of more aggressive compensation by considering only the bead distribution. If our baseline method compensates aggressively in a certain range, this is because most bead counts observed in that range are spillover counts. This approach highlights the allure and pitfalls of overcorrecting. While in Fig. 1 it may seem that spillR-naive compensates for all spillover by setting the first mode to zero (just like CATALYST), a closer inspection reveals that discrepancies between the bead spillover distribution and the first mode of the real cell distribution are not taken into account by either method, but do reflect in the compensation of spillR. A similar pattern can be seen in Panels A, B, and C of Fig. 3. This behavior reflects our assumption (A1) and highlights the role of bead experiments in the compensation process performed by spillR.

In the cell and bead data from CATALYST, our assumption (A1) seems to hold in some cases, but is violated in others. In our leading example in Fig. 1, we can see that the mode of the bead distribution of marker Yb173Di is very close to the mode in the cell data, supporting our assumption. In other cases however, the target marker mode differs more strongly between cell and bead data. We therefore recommend testing our assumptions against the available data and background knowledge. Comparing the target marker mode on cells and beads may serve as a first test for assumption (A1), and background knowledge on the markers may aid in assessing the plausibility of assumption (A2).

To understand and assess its applicability and performance, spillR offers a diagnostic plot (Fig. 1) of the spillover probability curve. We can judge if the curve makes sense by comparing it to the observed count and bead distributions. Methods based on non-negative least squares such as CATALYST are harder to diagnose as they minimize a cost function with no clear biological interpretation. In our view, one strength of spillR is that it does not assume a specific parametric model for count data. We believe that this is crucial because spillover compensation precedes many downstream analysis steps, and avoiding the introduction of bias is thus our priority.

In our experiments, we observe that the different methods may over- or undercompensate in different cases. The original experiment shown in Fig. 3 is designed to produce considerable spillover in the settings of the first column, and little spillover in the second column. Nonetheless, we observe weak and strong compensation by both spillR and CATALYST in both columns. Our diagnostic plots show overlap between the spillover marker distributions on beads and the first mode of the real cell distribution, thus the compensation by spillR is consistent with assumption (A1). The strong compensation performed by spillR-naive in both columns highlights the difficulty of balancing necessary and excessive compensation. spillR is designed to err on the side of preserving potentially meaningful patterns unless the bead distributions clearly suggest spillover. Overall, our results indicate that the performance of both spillR and CATALYST rests on the plausibility of their respective assumptions in the individual case. Since spillR relies on a different set of assumptions, it offers a complementary solution. Note that in principle it is also possible to combine the methods, e.g. by using spillR to identify spillover, and CATALYST to compensate for it.

Our basic method can also be applied to imaging mass cytometry (Angelo *et al.* 2014, Giesen *et al.* 2014, Bodenmiller 2016), although it will likely be beneficial to incorporate a spatial regularization term that enforces similarity between neighboring spillover estimates. We consider the design and evaluation of such an extension of our method a promising direction for future work.

## Supplementary Material

btae337_Supplementary_Data
