# Supplementary material for: spillR: spillover compensation in mass cytometry data
Source: Bioinformatics. 2024 Jun 6;40(6):btae337. doi: 10.1093/bioinformatics/btae337 (PMC11189660; doi:10.1093/bioinformatics/btae337)
Supplement: btae337_Supplementary_Data [file btae337_supplementary_data.pdf]

`spillR`: Spillover Compensation in Mass Cytometry Data

Supplementary Material

Marco Guazzini[1], Alexander G. Reisach[2], Sebastian Weichwald[3], and Christof Seiler[1,4,5]

[1]Department of Advanced Computing Sciences, Maastricht University, The Netherlands
[2]Université Paris Cité, CNRS, MAP5, F-75006 Paris, France
[3]Department of Mathematical Sciences, University of Copenhagen, Denmark
[4]Mathematics Centre Maastricht, Maastricht University, The Netherlands
[5]Center of Experimental Rheumatology, Department of Rheumatology,
University Hospital Zurich, University of Zurich, Switzerland

April 29, 2024

A EM Algorithm Example

Here we illustrate the procedure using a numerical example that includes one target and one spillover marker. We have one data matrix $\mathbf{Y}$ that contains real cell counts recorded for marker 1 (column 1) and the bead counts for marker 1 when the true marker was marker 2 (column 2). In practice, $\mathbf{Y}$ is usually a matrix with more than two columns representing multiple spillover markers. The index i is a specific cell in beads and real cells experiment, respectively. Let's assume the following counts,

$$\mathbf{Y} = (y_{ij}) = \begin{bmatrix} 3 & 2 \\ 5 & 3 \\ 17 & 2 \\ 3 & \\ 17 & \\ 2 & \end{bmatrix}.$$

```r
target    <- c(3, 5, 17,  3,  17, 2)
spillover <- c(2, 3,  2, NA, NA, NA)
Y = dplyr::bind_cols(target = target, spillover = spillover)
Y
```

```
## # A tibble: 6 x 2
##   target spillover
##    <dbl>     <dbl>
## 1      3         2
## 2      5         3
## 3     17         2
## 4      3        NA
## 5     17        NA
## 6      2        NA
```

- Initialization: We initialize our EM algorithm by estimating the conditional probability of observing y given that it belongs to the target marker, and another conditional probability given that it belongs to the spillover marker.

```
y_min <- min(Y$target)
y_max <- max(Y$target)
y_support <- y_min:y_max
fit1 <- density(Y$target, from = y_min, to = y_max)
fit2 <- density(Y$spillover, from = y_min, to = y_max, na.rm = TRUE)
f1 <- approxfun(fit1$x, fit1$y)
f2 <- approxfun(fit2$x, fit2$y)
P_Y1 <- f1(y_support)
P_Y1 <- P_Y1 / sum(P_Y1)
P_Y2 <- f2(y_support)
P_Y2 <- P_Y2 / sum(P_Y2)
P_YZ <- dplyr::bind_cols(P_Y1 = P_Y1, P_Y2 = P_Y2)
```

We initialize the mixture probabilities with the discrete uniform.

```
pi <- c(0.9, 0.1)
```

Now, we update these initial values using the E and M-steps.

- E-step: Calculate the posterior probability for the true marker, and the spillover marker.

```
P_ZY <- dplyr::mutate(P_YZ,
                      P_Y1 = pi[1] * P_Y1,
                      P_Y2 = pi[2] * P_Y2)
P_ZY <- P_ZY / rowSums(P_ZY)
P_ZY <- dplyr::bind_cols(target = y_support, P_ZY)
```

- M-step: Update the mixing probability vector,

```
n <- nrow(Y)
YP <- dplyr::left_join(Y, P_ZY, by = "target")
YP
```

```
## # A tibble: 6 x 4
##   target spillover  P_Y1     P_Y2
##    <dbl>     <dbl> <dbl>    <dbl>
## 1      3         2 0.717 2.83e- 1
## 2      5         3 1.00  5.70e-13
## 3     17         2 1     9.29e-17
## 4      3        NA 0.717 2.83e- 1
## 5     17        NA 1     9.29e-17
## 6      2        NA 0.550 4.50e- 1
```

```
pi <- c(sum(YP$P_Y1) / n, sum(YP$P_Y2) / n)
pi
```

```
## [1] 0.8307516 0.1692484
```

and re-estimate the distribution for the target marker using the posterior probabilities as weights, keep the non-target marker at its initial value,

```
fit1 <- density(Y$target, from = y_min, to = y_max, weights = YP$P_Y1)
f1 <- approxfun(fit1$x, fit1$y)
P_Y1 <- f1(y_support)
P_Y1 <- P_Y1 / sum(P_Y1)
P_YZ <- bind_cols(P_Y1 = P_Y1, P_Y2 = P_Y2)
```

and calculate the spillover probability estimate,

```
P_ZY <- dplyr::mutate(P_YZ,
                      P_Y1 = pi[1] * P_Y1,
                      P_Y2 = pi[2] * P_Y2)
P_ZY <- P_ZY / rowSums(P_ZY)
P_ZY <- dplyr::bind_cols(target = y_support, P_ZY)
P_ZY |>
  dplyr::mutate(p_spillover = round(1 - P_Y1, digits = 3)) |>
  dplyr::select(target, p_spillover) |>
  dplyr::filter(target %in% unique(Y$target))
```

```
##   target p_spillover
## 1      2       0.631
## 2      3       0.449
## 3      5       0.000
## 4     17       0.000
```

This is the result after one iteration.

B Generative Models

Bead Shift

Generative model for real cells Y of this experiment:

$$\begin{aligned}
I &\sim \text{Bernoulli}(0.1) && \text{(spillover indicator)}\\
Z &= I + 1 && \text{(channel number)}\\
(Y \mid Z = 1) &\sim \text{Poisson}(200) && \text{(target component)}\\
(Y \mid Z = 2) &\sim \text{Poisson}(70 + \tau) && \text{(spillover component with shift)}\\
Y &= (1 - I) \cdot (Y \mid Z = 1) + I \cdot (Y \mid Z = 2) && \text{(mixture).}
\end{aligned}$$

The generative model for beads is an independent copy of the unshifted $Y \mid Z = 2$ at $\tau = 0$.

Model Misspecification

Generative model for real cells Y of this experiment:

$$\begin{aligned}
I &\sim \text{Bernoulli}(0.1) && \text{(spillover indicator)}\\
Z &= I + 1 && \text{(channel number)}\\
T &\sim \text{Poisson}(200) && \text{(target)}\\
S &\sim \text{Poisson}(70) && \text{(spillover)}\\
M &\sim \text{Bernoulli}(\tau) && \text{(misspecification indicator)}\\
(Y \mid Z = 1) &= (1 - M) \cdot T + M \cdot S && \text{(target mixture component)}\\
(Y \mid Z = 2) &= (1 - M) \cdot S + M \cdot T && \text{(spillover mixture component)}\\
Y &= (1 - I) \cdot (Y \mid Z = 1) + I \cdot (Y \mid Z = 2) && \text{(mixture)}
\end{aligned}$$

The generative model for beads is an independent copy of $Y \mid Z = 2$.

Bimodal Spillover

Generative model for real cells Y of this experiment:

$$
\begin{aligned}
I &\sim \text{Bernoulli}(0.1) && \text{(spillover indictor)} \\
Z &= I + 1 && \text{(channel number)} \\
(Y \mid Z = 1) &\sim \text{Poisson}(200) && \text{(target component)} \\
H &\sim \text{Bernoulli}(\tau) && \text{(high count indicator)} \\
(S \mid H = 0) &\sim \text{Poisson}(70) && \text{(low count component)} \\
(S \mid H = 1) &\sim \text{Poisson}(330) && \text{(high count component)} \\
(Y \mid Z = 2) &= (1 - H) \cdot (S \mid H = 0) + H \cdot (S \mid H = 1) && \text{(spillover component)} \\
Y &= (1 - I) \cdot (Y \mid Z = 1) + I \cdot (Y \mid Z = 2) && \text{(mixture)}
\end{aligned}
$$

The generative model for beads is an independent copy of $Y \mid Z = 2$.
